# Diagnosis and prevalence of probable awake and sleep bruxism in adolescents: an exploratory analysis

**DOI:** 10.1590/0103-6440202305202

**Published:** 2023-07-17

**Authors:** Ivana Meyer Prado, Lucas Guimarães Abreu, Isabela Almeida Pordeus, Maryam Amin, Saul Martins Paiva, Junia Maria Serra-Negra

**Affiliations:** 1 Department of Pediatric Dentistry, Universidade Federal de Minas Gerais, Belo Horizonte, Minas Gerais, Brazil;; 2 Division of Pediatric Dentistry, University of Alberta, Edmonton, Alberta, Canada.

**Keywords:** bruxism, awake bruxism, epidemiology, adolescents.

## Abstract

The aims of this study were to perform an exploratory analysis of probable awake (AB) and sleep bruxism (SB) prevalence using of different diagnosis criteria based on the International Consensus; evaluate the associations between self-report and clinical signs/symptoms in adolescents. Participated in this cross-sectional study 403 adolescents aged 12- to 19-years-old enrolled in public and private schools from Belo Horizonte, Brazil. Parents/caregivers answered a questionnaire about sociodemographic status and adolescents’ health status. Adolescents answered a questionnaire evaluating AB (e.g., grinding and clenching) and SB (e.g., grinding, bracing, and thrusting) activities and frequent headaches. A clinical examination was performed on adolescents to evaluate bruxism clinical signs/symptoms (pain upon palpation on masseter and temporal, *linea alba*, indentation on the tongue and attrition wear severity). Descriptive statistics and Pearson’s Qui-square test were performed (P≤0.05). Adolescents mean age was 14.3±1.5 years, and 58.1% were female. Self-report of SB was identified in 31% of participants and self-report of AB in 51.6%. Almost all adolescents (99%) presented at least one tooth with attrition wear (98.5% on enamel and 0.5% on dentin), with a mean number of 12.4±5.7 teeth. Depending on the diagnosis criteria, the prevalence of probable SB and AB varied from 0- 99% and 0.2- 99%, respectively. A high inconsistency was found for the prevalence of probable AB and SB in adolescents, which were influenced by the different clinical sings/symptoms used as diagnosis criteria. Frequent headaches and pain upon palpation on masseter and temporal muscle were associated to self-report of AB and SB among adolescents.

## Resumo

Os objetivos deste estudo foram realizar uma análise exploratória da prevalência de provável bruxismo em vigília (BV)e do sono (BS) utilizando diferentes critérios de diagnóstico baseados no Consenso Internacional; avaliar a associação entre o autorrelato e os sinais/sintomas clínicos em adolescentes. Participaram deste estudo transversal 403 adolescentes de 12 a 19 anos de idade matriculados em escolas públicas e privadas de Belo Horizonte, Brasil. Os pais/responsáveis responderam um questionário sobre dados sociodemográficos e estado de saúde dos adolescentes. Os adolescentes relataram atividades do BV (ranger e apertar) e BS (ranger, *bracing* e *thrusting*) e dores de cabeça frequentes. Um exame clínico foi realizado nos adolescentes para avaliar os sinais/sintomas clínicos do bruxismo (dor à palpação nos músculos masseter e temporal, marcas de endentação na língua e linha alba, desgaste dentário por atrição). Análises descritiva e teste Qui-quadrado de Pearson foram realizados (P≤0,05). A média de idade dos adolescentes foi de 14,3±1,5 anos e 58,1% eram do sexo feminino. O autorrelato de BS foi identificado em 31% dos participantes e o autorrelato do BV em 51,6%. Quase a totalidade dos adolescentes (99%) apresentaram pelo menos um dente com desgaste dentário (98,5% em esmalte; 0,5% em dentina), com média de 12,4±5,7 dentes acometidos. Dependendo do critério de diagnóstico, a prevalência do provável BS e BV variou de 0- 99% e 0,2- 99%, respectivamente. Uma grande inconsistência foi identificada na prevalência de provável BV e BS em adolescentes, que foram influenciadas pelos diferentes sinais/sintomas usados como critério de diagnóstico. Dores de cabeça frequentes e dor à palpação no masseter e temporal foram associados ao autorrelato de bruxismo em crianças e adolescentes.

## Introduction

Bruxism is a masticatory muscle activity that affects all age groups, from children to the elderly ^1^ ^,^ ^2^. When it occurs during sleep (sleep bruxism), it is characterized as a rhythmic (phasic) or non-rhythmic (tonic) muscular activity, while awake bruxism is characterized by repetitive or sustained tooth contact and/or by bracing or thrusting of the mandible during wakefulness ^1^. Bruxism etiology is multifactorial. In children and adolescents, it is associated with sex, sleep features (e.g., quality, duration, disturbances), psychological factors (e.g., stress, anxiety, depressive mood, personality traits), respiratory disorders, snoring, and smoking ^3^ ^,^ ^4^. 

According to the International Consensus, the clinical features of awake and sleep bruxism are masticatory muscle hypertrophy, indentation marks on the tongue and/or *linea alba* on the inner cheek, damage to the dental tissue, repetitive failures of restorations or mechanical tooth wear (dental attrition) ^1^. In children, primary canine wear, dental wear, and headaches are the most prevalent clinical signs/symptoms of sleep bruxism ^5^, and facial pain is associated with both awake and sleep bruxism in adolescents ^4^. 

Bruxism is graded as: ‘possible’, when it is based on a positive self-report; ‘probable’, when it is based on a positive clinical inspection, despite a positive self-report; and ‘definite’, when it is based on a positive instrumental evaluation, despite a positive clinical inspection and/or self-report ^1^. Most studies in children and adolescents rely on parental- or self-report ^2^ ^,^ ^3^, and some recent studies rely on the report plus the presence of clinical features ^5^ ^,^ ^6^. The lack of standardized diagnostic methods leads to a high prevalence variability of the behavior, ranging from 3% to 49% in children and adolescents ^2^. Also, there are fewer studies focused on adolescents, and research with young individuals usually does not separate adolescents from children when analyzing or reporting data ^3^ ^,^ ^7^, making it difficult to understand the epidemiology and impact of bruxism in this specific period of life, that brings several physical and emotional changes ^8^. In the absence of a standardized diagnosis criterion, and the impossibility of comparing studies, it is hard to provide good scientific evidence for clinicians and healthcare professionals.

Despite advances in the definition, classification, and management of bruxism through the recent version of the International Consensus, this publication focusses mainly on adult population, with children being mentioned only to state that, for them, ‘possible’ bruxism evaluation should rely on parental-report ^1^. It is still unknown if both awake and sleep bruxism share the same clinical features or not. Furthermore, it is not clear which or how many clinical signs/symptoms should be considered to determine a ‘probable’ bruxism occurrence ^1^. The lack of robust epidemiological evidence when it comes to adolescent’s points out the need to verify the prevalence of clinical features in this population, as well as the prevalence of probable bruxism based on the recent Consensus proposal. Based on the aforementioned, the aims of this study were to perform an exploratory analysis to evaluate different prevalence estimates of probable awake and sleep bruxism considering different diagnosis criteria according with the International Consensus of bruxism; also, evaluate the association of self-reported awake and sleep bruxism activities and clinical features of bruxism. The null hypothesis of the study is that there are no differences in prevalence estimates of probable awake and sleep bruxism considering different diagnosis criteria, and there are no associations between self-reported awake and sleep bruxism activities and clinical features of bruxism in adolescents.

## Materials and methods

This study was reported following the STROBE Checklist for cross-sectional studies ^9^.

### Ethical aspects

This study was conducted by the principles stated in the Declaration of Helsinki (revised in World Medical Association 2013). Ethical approval was obtained from the Human Research Ethics Committee of the Federal University of Minas Gerais (protocol #82839718.4.0000.5149). All parents/caregivers and adolescents were informed about the objectives of the research, the method employed, and the voluntary nature of the study. Assurance of confidentiality was also guaranteed to participants.

### Study design, setting, and participants

This cross-sectional population-based study was conducted with 450 adolescents from Belo Horizonte between September and December 2018. The city is located in the southeast region of Brazil and is divided into nine administrative regions. Adolescents were randomly selected in a multiple-stage sampling method. One public and one private school from each of the nine regions were randomly selected to participate in the study. Afterward, one classroom from each of the 18 schools across the regions was randomly selected and all adolescents from those classrooms were invited to participate in the study.

For participation, inclusion criteria were applied to adolescents as follows: age between 12 and 19 years and enrollment in a private or a public school of Belo Horizonte. Exclusion criteria were adolescents using antidepressant and/or anticonvulsant medication ^10^, as well as adolescents with syndromes and/or cognitive disorders. The participant or their parents/caregivers provided information on adolescents’ health and medication use. The age between 12 and 19 years old was selected based on the classification of adolescence by the World Health Organization ^8^ and considering that at the age of 12, all permanent teeth, except the third molars, have already erupted in the oral cavity ^11^. All adolescents and their parents/caregivers signed an informed consent form to participate in the study.

### Non-clinical data collection

Parent’s/caregiver’s questionnaire

Parents/caregivers answered a questionnaire regarding adolescents’ age, health status (chronic conditions, syndromes, and/or cognitive disorders), and medication use ^6^.

### Adolescents’ questionnaire

Adolescents answered a questionnaire built by the research group for evaluation of the occurrence of awake bruxism (grinding and clenching activity), sleep bruxism (grinding and thrusting/bracing activity), and history of frequent headaches. The questionnaire was created based on recommendations and methods of previous studies ^1^ ^,^ ^3^ ^,^ ^6^. The questions were as follows:

1. Awake bruxism - grinding activity: Did you grind your teeth while awake over the past two weeks?
2. Awake bruxism - clenching activity: Did you clench your teeth while awake over the past two weeks?
3. Sleep bruxism - grinding activity: Has anyone told you or are you aware of the fact that you grind your teeth during a sleep over the past two weeks or that you grind your teeth during sleep?
4. Sleep bruxism - thrusting activity: When awakening in the morning or waking up at night, over the past two weeks, have you had your jaw positioned forward or sideways?
5. Sleep bruxism - bracing activity item: When awakening in the morning or waking up at night, over the past two weeks, have you had your jaw in a steady/rigid position (with difficulty in opening your mouth)?
6. Frequent headaches: Did you have headaches often (more than three times a week), in the past two weeks?

Questions 1 to 5 could be answered with “no”, “sometimes” or “often”. Question 6 could be answered with “no” or “yes”.

### Clinical data collection

After the questionnaire application, a clinical examination was performed by one trained researcher using appropriate equipment for individual protection, mouth mirrors (Prisma®, PRISMA Instrumentos Odontológicos Ltda., São Paulo, Brazil), dental gauze, and a headlamp (PETZL, PETZL Technical Institute, Salt Lake City, USA). An assistant wrote down the collected information. For the clinical examination, all adolescents were seated on a chair in front of the trained researcher.

Based on the clinical signs/symptoms of bruxism stated by International Consensus on the assessment of bruxism ^1^, the following clinical signs/symptoms were evaluated based on instruments and methods published previously: pain upon palpation in the masseter muscles, pain upon palpation in the temporal muscles, indentation marks on the tongue, and *linea alba* on the inner cheek. Those clinical features were considered as ‘present’ or ‘absent’. Pain upon palpation was evaluated by applying force with the index and the middle finger bilaterally on the masseter and temporal muscles while inquiring the adolescent if he/she felt pain ^12^. Tooth wear facets due to dental attrition on each tooth were also assessed ^13^. Tooth wear was evaluated based on a five-point ordinal grading scale for occlusal/incisal assessment (0=no wear; 1=visible wear within the enamel; 2=visible wear with dentin exposure and loss of clinical crown height of ≤ 1/3; 3=loss of crown height > 1/3 but < 2/3; and 4=loss of crown height ≥ 2/3. Deciduous teeth and teeth with extensive caries lesions or extensive restorations were excluded from the statistical analysis.

### Training and calibration Exercise

One examiner (IMP) underwent a calibration process with two phases, conducted by a clinical expert. The first phase consisted of photograph analysis and discussion of the criteria used to identify tooth wear facets due to dental attrition (intrinsic mechanical tooth wear) and the clinical differences between tooth wear by dental attrition compared to tooth wear by dental abrasion and dental erosion ^6^. Tooth wear due to dental attrition was evaluated based on a five-point ordinal grading scale for occlusal/incisal assessment ^13^.

The second phase occurred one week after the first phase and consisted of a clinical examination of ten adolescents to identify tooth wear due to dental attrition. Inter-examiner agreement was determined based on the results of the comparison between the assessment of the clinical expert and the assessment of the examiner. All adolescents were re-examined in a fortnight’s time to determine the intra-examiner agreement. Kappa coefficient values for inter-examiner (Kappa = 0.85) and intra-examiner (Kappa = 0.80) showed substantial agreement ^14^.

The examiner was also trained by the clinical expert to perform the evaluation of other clinical signs and symptoms of bruxism: pain upon palpation in the masseter, pain upon palpation in the temporal muscles, indentation marks on the tongue, and *linea alba* on the inner cheek ^1^.

### Study variables

The variables representative of self-reported sleep bruxism activity were: “self-report of sleep bruxism - grinding activity”, “self-report of sleep bruxism - thrusting activity”, and “self-report of sleep bruxism - bracing activity”. Based on the combination of the answers of those three variables, the variable “possible sleep bruxism” was created. When the same participant reported more than one activity of sleep bruxism, the higher frequency of occurrence (often) was considered. The variables representing self-reported awake bruxism activity were: “self-report of awake bruxism - grinding activity” and “self-report of awake bruxism - clenching activity”. The variable “possible awake bruxism” was created based on the combination of the answers of those two variables. When the same adolescent reported more than one activity of awake bruxism, the higher frequency of occurrence (often) was taken into account.

The variables “possible sleep bruxism” and “possible awake bruxism” were ordinal (“no activity”, “sometimes” or “often”). The variables “pain upon palpation on temporal muscle”, “pain upon palpation on masseter muscle”, “indentation marks on the tongue”, and “*linea alba*” were dichotomous (“present” or “absent”). The variable “frequent headaches” was also dichotomous (“yes” or “no”). The variables related to tooth wear due to dental attrition (“number of teeth with attrition wear”, “number of anterior teeth with attrition wear”, “number of posterior teeth with attrition wear”, and “attrition wear score”) were discrete quantitative variables. For the creation of the variable “attrition wear score”, information on the teeth with the worse attrition wear score for each adolescent was computed.

The prevalence of “provable sleep bruxism” and “probable awake bruxism” were verified based on different diagnosis criteria, according to the International Consensus ^1^, considering positive self-report along with the presence of different clinical signs and symptoms and considering only the presence of different clinical signs and symptoms of bruxism.

### Sample size

Sample size was calculated based on a population proportion estimation formula, using the following parameters: 95% confidence interval, a 4% standard error, and a 15.3% prevalence of possible sleep bruxism ^15^. No published study evaluating the prevalence of probable sleep bruxism among 12- to 19-year-old adolescents were found in the literature until the beginning of the present study. Therefore, the prevalence estimate of the most similar study ^15^ was used for the sample size calculation herein. The parameters estimated a sample size of 311 adolescents. Due to a two-stage cluster sampling (first the random selection of schools, second the random selection of classrooms) an increase of 10% for each stage was necessary in order to increase sample precision and minimize possible sample bias. Therefore, a correction factor of 1.2 was applied in the sample size estimation of 311, which resulted in a minimum sample size of 373 adolescents. Finally, an increase of 20% due to possible losses was also applied, and the final sample size was calculated in 448 adolescents.

A total of 450 adolescents were invited to participate in the study, and 403 were included. The other 47 (10.4%) were excluded because they either failed to complete all instruments or were users of antidepressant/anticonvulsant medications. Adolescents' mean age was 14.3±1.5 years and most participants were females (58.1%).

### Pilot study

Forty-four adolescents, approximately 10% of the final sample, enrolled in a public school of Belo Horizonte not included in the main study, were randomly selected to participate in a pilot study. The pilot study included adolescents aged 12 to 19 years old and aimed to test the proposed method as well as whether adolescents had understood the questions from the questionnaire. After getting adolescents’ parents’/caregivers’ consent and their own consent to participate, adolescents answered the questionnaire in a classroom in the school. After answering the questionnaire, they were clinically examined individually in a separate classroom. After data collection, researchers concluded that changes in the methods of the main study were unnecessary. Adolescents who had participated in this pilot study were not included in the main study sample.

### Statistical methods

All data were organized and analyzes using the Statistical Package for the Social Sciences program for windows (SPSS Inc., Chicago IL, USA - Version 21.0). Descriptive statistics were performed to evaluate the frequency, mean, median, standard deviation, and range of studied variables and evaluate the different percentages of probable awake bruxism and probable sleep bruxism based on different diagnosis criteria ^1^. Bivariate analysis using Pearson’s Chi-Square test, Fisher’s Exact test, and Kruskal-Wallis were performed to compare the association between the clinical signs/symptoms and adolescents’ report of bruxism activity (P ≤ 0.05).

## Results

A total of 125 adolescents (31%) reported at least one of the sleep bruxism activities. When considering awake bruxism, the prevalence of self-report of grinding and/or clenching activity was 51.6% (208). Almost all adolescents (99%) presented at least one tooth with attrition wear (98.5% on enamel and 0.5% on dentin), with a mean number of 12.4 (±5.7) teeth with attrition. Frequent headaches were reported by 38.7% and 41.4% of adolescents had *linea* alba (Table 1).

**Table 1:** Descriptive statistics of bruxism activity and clinical signs and symptoms among adolescents from Belo Horizonte, Brazil.

Variables	Frequency (%)
Sleep Bruxism	
Self-report of Sleep Bruxism - Grinding activity	
No activity	313 (77.7)
Sometimes	65 (16.1)
Often	25 (06.2)
Self-report of Sleep Bruxism - Thrusting activity	
No activity	345 (85.6)
Sometimes	44 (10.9)
Often	14 (03.5)
Self-report of Sleep Bruxism - Bracing activity	
No activity	353 (87.6)
Sometimes	43 (10.7)
Often	07 (01.7)
Possible Sleep Bruxism*	
No activity	278 (69,0)
Sometimes	89 (22,1)
Often	36 (08,9)
Awake Bruxism	
Self-report of Awake Bruxism - Grinding activity	
No activity	214 (53.1)
Sometimes	140 (34.7)
Often	49 (12.2)
Self-report of Awake Bruxism - Clenching activity	
No activity	303 (75.2)
Sometimes	77 (19.1)
Often	23 (05.7)
Possible Awake Bruxism^┼^	
No activity	195 (48.4)
Sometimes	150 (37.2)
Often	58 (14.4)
Clinical Signs and Symptoms	
Frequent headaches (3 or more times a week)		
No	247 (61.3)
Yes	156 (38.7)
Pain upon palpation on temporal muscle	
Absent	359 (89.1)
Present	44 (10.9)
Pain upon palpation on masseter muscle	
Absent	337 (83.6)
Present	66 (16.4)
Indentation marks on the tongue	
Absent	310 (76.9)
Present	93 (23.1)
*Linea alba*	
Absent	236 (58.6)
Present	167 (41.4)
Number of teeth with attrition wear	
Mean [± SD]	12.4 [± 5.7]
Median [Min - Max]	12 [0 - 28]
Number of anterior teeth with attrition wear	
Mean [± SD]	6.3 [± 3.4]
Median [Min - Max]	6 [0 - 12]
Number of posterior teeth with attrition wear	
Mean [± SD]	6.1 [± 3.8]
Median [Min - Max]	6 [0 - 16]
Attrition wear score^╪^	
0	04 (01.0)
1	397 (98.5)
2	02 (0.5)

SD = Standard deviation; Min = Minimum; Max = Maximum.

*Prevalence considering all tree activities of sleep bruxism (grinding, thrusting, and bracing); ┼Prevalence considering both activities of awake bruxism (grinding and clenching); ^╪^The tooth with the worse score was considered in the analysis.

The prevalence of frequent headaches and pain upon palpation on masseter and temporal muscles were higher among adolescents with positive self-report of sleep bruxism - grinding activity (P<.001), self-report of sleep bruxism - bracing activity (P≤.004), self-report of awake bruxism - grinding activity (P<.001), and self-report of awake bruxism - clenching activity (P<.001). *Linea* alba (P=.009) and pain upon palpation on masseter and temporal muscles (P≤.001) were more prevalent among adolescents with self-report of sleep bruxism - thrusting activity (Table 2 and Table 3).

Frequent headaches and pain upon palpation on masseter and temporal muscles were more prevalent among adolescents with positive self-report of all activities of sleep bruxism combined (“possible sleep bruxism”) (P<.001). The same result was found when all activities of sleep bruxism were considered combined (“possible awake bruxism”) (p<.001) (Table 4).

Table 5 displays the prevalence values of probable sleep bruxism based on different diagnose criteria. The prevalence of probable sleep bruxism varied from 0% to 99.8%, depending on the diagnosis criteria. When self-report of sleep bruxism was considered (positive possible sleep bruxism) along with the presence of any of all clinical signs and symptoms of bruxism, the prevalence of probable sleep bruxism was 31%, which is the same prevalence of possible sleep bruxism. When probable sleep bruxism was diagnosed based on the presence of any of all clinical signs and symptoms of bruxism despite a positive self-report (positive possible sleep bruxism), the prevalence was 99.8%.

Table 6 displays the prevalence values of probable awake bruxism based on different diagnose criteria. The prevalence of probable awake bruxism varied from 0.2% to 99.8%, depending on the diagnosis criteria. When self-report of awake bruxism was considered (positive possible awake bruxism) along with the presence of any of all clinical signs and symptoms of bruxism, the prevalence of probable awake bruxism was 51.4%, the same prevalence of possible awake bruxism. When probable awake bruxism was diagnosed based on the presence of any of all clinical signs and symptoms of bruxism despite a positive self-report (positive possible awake bruxism), the prevalence was 99.8%.

## Discussion

There was a high variability in the prevalence of probable sleep and awake bruxism based on different diagnosis criteria according with the International Consensus ^1^, allowing us to reject the null hypothesis of the study. The lowest prevalence was identified when the diagnosis of probable sleep and awake bruxism were based on a positive self-report of bruxism along with presence of attrition wear within the dentin, while the highest prevalence was identified when the diagnosis of probable bruxism was based on the presence of tooth wear on enamel or dentin, regardless of a positive self-report. Almost all adolescents exhibited at least one tooth with mild wear (within the enamel) and only two-exhibited moderate wear (within the dentin). Another Brazilian study identified mild tooth wear in 84.4% of 12-year-old adolescents ^6^. Prevalence of moderate and severe tooth wear in at least one tooth was 24% in 12- to 17-year-old adolescents from the United States ^16^. Tooth wear has been acknowledged as one of the clinical signs/symptoms of bruxism, being a parameter used for its diagnosis ^6^. However, evidence regarding its prevalence and association with bruxism activity in the mixed and permanent dentition of adolescents is scarce. It is possible that at such a young age, only a small percentage of adolescents will exhibit severe attrition wear ^16^, indicating that this clinical feature is not an accurate sign of bruxism activity in this population. Further investigation is necessary to clarify whether attrition wear is associated to different activities of awake and sleep bruxism in adolescents and whether it should be taken into account for bruxism diagnosis in this population. 

**Table 2:** Bivariate analysis of Self-reported Sleep Bruxism and clinical signs and symptoms among adolescents from Belo Horizonte, Brazi

Clinical signs	Self-report of Sleep Bruxism - Grinding activity			P	Self-report of Sleep Bruxism - Thrusting activity			P	Self-report of Sleep Bruxism - Bracing activity			P
	No	Sometimes	Often		No	Sometimes	Often		No	Sometimes	Often	
Headaches frequently (3 or more times a week)												
Yes	103 (32.9)^a^	34 (52.3)^b^	19 (76.0)^b^	<.001^*^	126 (36.5)	24 (54.5)	06 (42.9)	.064^*^	126 (35.7)^a^	26 (60.5)^b^	04 (57.1)^b^	.004^┼^
No	210 (67.1)	31 (47.7)	06 (24.0)		219 (63.5)	20 (45.5)	08 (57.1)		227 (64.3)	17 (39.5)	03 (42.9)	
Pain upon palpation on temporal muscle												
Present	18 (05.8)^a^	17 (26.2)^b^	09 (36.0)^b^	<.001^*^	30 (08.7)^a^	08 (18.2)^a,b^	06 (42.9)^b^	.001^┼^	28 (07.9)^a^	14 (32.6)^b^	02 (28.6)^a,b^	<.001^┼^
Absent	295 (94.2)	48 (73.8)	16 (64.0)		315 (91.3)	36 (81.8)	08 (57.1)		325 (92.1)	29 (67.4)	05 (71.4)	
Pain upon palpation on masseter muscle												
Present	32 (10.2)^a^	27 (41.5)^b^	07 (28.0)^b^	<.001^┼^	40 (11.6)^a^	19 (43.2)^b^	07 (50.0)^b^	<.001^┼^	45 (12.7)^a^	15 (34.9)^b^	06 (85.7)^c^	<.001^┼^
Absent	281 (89.8)	38 (58.5)	18 (05.3)		305 (88.4)	25 (56.8)	07 (50.0)		308 (87.3)	28 (65.1)	01 (14.3)	
Indentations on the tongue												
Present	72 (23.0)	17 (26.2)	04 (16.0)	.593^*^	77 (22.3)	13 (29.5)	03 (21.4)	.558^┼^	81 (22.9)	10 (23.3)	02 (28.6)	.900^┼^
Absent	241 (77.0)	48 (73.8)	21 (84.0)		268 (77.7)	31 (70.5)	11 (78.6)		272 (77.1)	33 (76.7)	05 (71.4)	
Linea alba												
Present	128 (40.9)	31 (47.7)	08 (32.0)	.380^*^	152 (44.1)^a^	09 (20.5)^b^	06 (42.9)^a,b^	.009^*^	149 (42.2)	14 (32.6)	04 (57.1)	.346^┼^
Absent	185 (59.1)	34 (52.3)	17 (68.0)		193 (55.9)	35 (79.5)	08 (57.1)		204 (57.8)	29 (67.4)	03 (42.9)	
Attrition wear score												
0	04 (01.3)	0 (0.0)	0 (0.0)	1.000^┼^	04 (01.2)	0 (0.0)	0 (0.0)	1.000^┼^	03 (0.8)	01 (02.3)	0 (0.0)	.551^┼^
1	307 (98.1)	65 (100)	25 (100)		339 (98.3)	44 (100)	14 (100)		348 (98.6)	42 (97.7)	07 (100)	
2	02 (0.6)	0 (0.0)	0 (0.0)		02 (0.6)	0 (0.0)	0 (0.0)		02 (0.6)	0 (0.0)	0 (0.0)	
Number of teeth with attrition wear												
Mean [± SD]	12.2 [±5.9]	12.8 [±5.3]	14.0 [±4.3]	.182^╪^	12.4 [±5.8]	12.5 [±5.4]	11.5 [±5.0]	.861^╪^	12.5 [±5.9]	11.9 [±5.0]	11.0 [±4.3]	.741^╪^
Median [Min-Max]	12 [0-28]	13 [3-24]	14 [5-22]		12 [0-28]	12 [2-24]	12 [4-20]		12 [0-28]	13 [0-25]	12 [6-17]	
Number of anterior teeth with attrition wear												
Mean [± SD]	6.0 [±3.4]^a^	7.0 [±3.5]^a^	7.8 [±3.0]^a^	.010^╪^	6.3 [±3.5]	6.3 [±3.3]	6.4 [±3.8]	.986^╪^	6.2 [±3.4]	6.8 [±3.6]	6.1 [±3.3]	.585^╪^
Median [Min-Max]	6 [0-12]	8 [0-12]	7 [2-12]		6 [0-12]	6 [0-12]	5.5 [0-12]		6 [0-12]	7 [0-12]	5 [2-12]	
Number of posterior teeth with attrition wear												
Mean [± SD]	6.1 [±4.0]	5.7 [±3.4]	6.2 [±3.1]	.781^╪^	6.1 [±3.8]	6.2 [±4.0]	5.1 [±2.8]	.688^╪^	6.2 [±3.9]	5.1 [±3.6]	4.8 [±3.1]	.130^╪^
Median [Min-Max]	6 [0-16]	5 [0-13]	6 [1-12]		6 [0-16]	6.5 [0-15]	5 [1-9]		6 [0-16]	4 [0-13]	5 [1-9]	

* Pearson’s Qui-Square test and post-tests; ^┼^Fisher’s Exact test and post-tests; ^╪^Kruskal-Wallis test and post-tests. P = Probability value; SD = Standard Deviation; Min = Minimum; Max = Maximum. Values in parenthesis represent the percentage on the column. Values in bold represent statistically significant association. Different letters represent statistical differences.

**Table 3:** Bivariate analysis of Self-reported Awake Bruxism and clinical signs and symptoms among adolescents from Belo Horizonte, Brazil.

Clinical signs	Self-report of Awake Bruxism - Grinding activity			P	Self-report of Awake Bruxism -Clenching activity			P
	No	Sometimes	Often		No	Sometimes	Often	
Headaches frequently (3 or more times a week)								
Yes	103 (34.0)^a^	36 (46.8)^a,b^	17 (73.9)^b^	<.001^*^	62 (29.0)^a^	63 (45.0)^b^	31 (63.3)^b^	<.001^*^
No	200 (66.0)	41 (53.2)	06 (26.1)		152 (71.0)	77 (55.0)	18 (36.7)	
Pain upon palpation on temporal muscle								
Present	22 (07.3)^a^	14 (18.2)^b^	08 (34.8)^b^	<.001^┼^	15 (07.0)^a^	13 (09.3)^a^	16 (32.7)^b^	<.001^*^
Absent	281 (92.7)	63 (81.8)	15 (65.2)		199 (93.0)	127 (90.7)	33 (67.3)	
Pain upon palpation on masseter muscle								
Present	32 (10.6)^a^	25 (32.5)^b^	09 (39.1)^b^	<.001^┼^	17 (07.9)^a^	28 (20.0)^b^	21 (42.9)^c^	<.001^*^
Absent	271 (89.4)	52 (67.5)	14 (60.9)		197 (92.1)	112 (80.0)	28 (57.1)	
Indentations on the tongue								
Present	74 (24.4)	17 (22.1)	02 (08.7)	.219^*^	48 (22.4)	34 (24.3)	11 (22.4)	.916^*^
Absent	229 (75.6)	60 (77.9)	21 (91.3)		166 (77.6)	106 (75.7)	38 (77.6)	
Linea alba								
Present	124 (40.9)	33 (42.9)	10 (43.5)	.915^*^	85 (39.7)	61 (43.6)	21 (42.9)	.755^*^
Absent	179 (59.1)	44 (57.1)	13 (56.5)		129 (60.3)	79 (56.4)	28 (57.1)	
Attrition wear score								
0	04 (01.3)	0 (0.0)	0 (0.0)	.816^┼^	02 (0.9)	02 (01.4)	0 (0.0)	.923^┼^
1	297 (98.0)	77 (100)	23 (100)		211 (98.6)	137 (97.9)	49 (100)	
2	02 (0.7)	0 (0.0)	0 (0.0)		01 (0.5)	01 (0.4)	0 (0.0)	
Number of teeth with attrition wear								
Mean [± SD]	12.2 [±5.9]	12.9 [±5.3]	13.8 [±4.6]	.185^╪^	12.4 [±5.9]	12.2 [±5.7]	13.1 [±5.1]	.554^╪^
Median [Min-Max]	12 [0-28]	13 [3-23]	13 [3-22]		12 [0-28]	12 [0-25]	13 [4-22]	
Number of anterior teeth with attrition wear								
Mean [± SD]	6.1 [±3.5]	6.7 [±3.2]	7.2 [±3.4]	.171^╪^	6.2 [±3.4]	6.2 [±3.6]	6.9 [±3.2]	.401^╪^
Median [Min-Max]	6 [0-2]	7 [0-12]	7 [0-12]		6 [0-12]	6.5 [0-12]	7 [0-12]	
Number of posterior teeth with attrition wear								
Mean [± SD]	6.0 [±3.9]	6.1 [±3.7]	6.6 [±3.7]	.765^╪^	6.2 [±4.0]	5.9 [±3.4]	6.1 [±4.2]	.953^╪^
Median [Min-Max]	6 [0-16]	6 [0-15]	6 [0-14]		6 [0-16]	6 [0-14]	6 [0-15]	

Pearson’s Qui-Square test and post-tests; ^┼^Fisher’s Exact test and post-tests; ^╪^Kruskal-Wallis test and post-tests. P = Probability value; SD = Standard Deviation; Min = Minimum; Max = Maximum. Values in parenthesis represent the percentage on the column. Values in bold represent statistically significant association. Different letters represent statistical differences.

**Table 4:** Bivariate analysis of Possible Awake Bruxism, Possible Sleep Bruxism and clinical signs and symptoms among adolescents from Belo Horizonte, Brazil

Clinical signs	Possible Sleep bruxism			P	Possible Awake Bruxism			P
	No	Sometimes	Often		No	Sometimes	Often	
Headaches frequently (3 or more times a week)								
Yes	85 (30.6)^a^	48 (53.9)^b^	23 (63.9)^b^	<.001^*^	56 (28.7)^a^	62 (41.3)^b^	38 (65.5)^c^	<.001^*^
No	193 (69.4)	41 (46.1)	13 (36.1)		139 (71.3)	88 (58.7)	20 (34.5)	
Pain upon palpation on temporal muscle								
Present	15 (05.4)^a^	17 (19.1)^b^	12 (33.3)^b^	<.001^┼^	11 (05.6)^a^	15 (10.0)^a^	18 (31.0)^b^	<.001^*^
Absent	263 (94.6)	72 (80.9)	24 (66.7)		184 (94.4)	135 (90.0)	40 (69.0)	
Pain upon palpation on masseter muscle								
Present	23 (08.3)^a^	29 (32.6)^b^	14 (38.9)^b^	<.001^*^	12 (06.2)^a^	30 (20.0)^b^	24 (41.4)^c^	<.001^*^
Absent	255 (91.7)	60 (67.4)	22 (61.1)		183 (93.8)	120 (80.0)	34 (58.6)	
Indentations on the tongue								
Present	63 (22.7)	23 (25.8)	07 (19.4)	.699^*^	44 (22.6)	37 (24.7)	12 (20.7)	.803^*^
Absent	215 (77.3)	66 (74.2)	29 (80.6)		151 (77.4)	113 (75.3)	46 (79.3)	
Linea alba								
Present	120 (43.2)	35 (39.3)	12 (33.3)	.467^*^	77 (39.5)	67 (44.7)	23 (39.7)	.605^*^
Absent	158 (56.8)	54 (60.7)	24 (66.7)		118 (60.5)	83 (55.3)	35 (60.3)	
Attrition wear score								
0	03 (01.1)	01 (01.1)	0 (0.0)	1.000^┼^	02 (01.0)	02 (01.3)	0 (0.0)	1.000^┼^
1	273 (98.2)	88 (98.9)	36 (100)		192 (98.5)	147 (98.0)	58 (100)	
2	02 (0.7)	0 (0.0)	0 (0.0)		01 (0.5)	01 (0.7)	0 (0.0)	
Number of teeth with attrition wear								
Mean [± SD]	12.3 [±5.9]	12.5 [±5.5]	13.0 [±4.9]	.709^╪^	12.3 [±6.0]	12.4 [±5.7]	12.8 [±5.0]	.700^╪^
Median [Min-Max]	12 [0-28]	13 [0-25]	13 [4-22]		12 [0-28]	12 [0-25]	13 [3-22]	
Number of anterior teeth with attrition wear								
Mean [± SD]	6.1 [±3.4]	6.7 [±3.6]	7.1 [±3.4]	.106^╪^	6.1 [±3.4]	6.3 [±3.5]	6.7 [±3.3]	.455^╪^
Median [Min-Max]	6 [0-12]	7 [0-12]	7 [0-12]		6 [0-12]	7 [0-12]	7 [0-12]	
Number of posterior teeth with attrition wear								
Mean [± SD]	6.2 [±3.9]	5.8 [±3.8]	5.8 [±3.0]	.629^╪^	6.2 [±4.0]	6.0 [±3.5]	6.5 [±4.0]	.959^╪^
Median [Min-Max]	6 [0-16]	5 [ 0-15]	6 [1-12]		6 [0-16]	6 [0-14]	5.5 [0-15]	

Pearson’s Qui-Square test and post-tests; ^┼^Fisher’s Exact test and post-tests; ^╪^Kruskal-Wallis test and post-tests. P = Probability value; SD = Standard Deviation; Min = Minimum; Max = Maximum. Values in parenthesis represent the percentage on the column. Values in bold represent statistically significant association. Different letters represent statistical differences.

**Table 5:** Prevalence of Probable Sleep Bruxism in adolescents based on different diagnose criteria.

Variables	Frequency (%)
Possible Sleep Bruxism	
Positive	125 (31.0)
Negative	278 (69.0)
Probable Sleep Bruxism based on positive possible sleep bruxism + presence of clinical signs and symptoms (frequent headaches or pain upon palpation on masseter or pain upon palpation on temporal or tooth wear on enamel or tooth wear on dentin or indentation marks on the tongue or *line alba*)	
Positive	125 (31.0)
Negative	278 (69.0)
Probable Sleep Bruxism based on positive possible sleep bruxism + presence of clinical signs and symptoms of bruxism (frequent headaches or masseter pain upon palpation or temporal pain upon palpation or tooth wear on dentin or indentation marks on the tongue or *line alba*)	
Positive	109 (27.0)
Negative	294 (73.0)
Probable Sleep Bruxism based on positive possible sleep bruxism + pain upon palpation on masseter or temporal muscles	
Positive	54 (13.4)
Negative	349 (86.6)
Probable Sleep Bruxism based on positive possible sleep bruxism + frequent headaches	
Positive	71 (17.6)
Negative	332 (82.4)
Probable Sleep Bruxism based on positive possible sleep bruxism + tooth wear on enamel or dentin	
Positive	124 (30.8)
Negative	279 (69.2)
Probable Sleep Bruxism based on positive possible sleep bruxism + tooth wear on dentin	
Positive	0 (0.0)
Negative	403 (100)
Probable Sleep Bruxism based on positive possible sleep bruxism + indentation marks on the tongue or *linea alba*	
Positive	59 (14.6)
Negative	344 (85.4)
Probable Bruxism based on clinical signs and symptoms of bruxism (frequent headaches or pain upon palpation on masseter or pain upon palpation on temporal or tooth wear on enamel or tooth wear on dentin or indentation marks on the tongue or *line alba*), despite a positive self-report	
Positive	402 (99.8)
Negative	01 (00.2)
Probable Bruxism based on clinical signs and symptoms of bruxism (frequent headaches or pain upon palpation on masseter or pain upon palpation on temporal or tooth wear on dentin or indentation marks on the tongue or *line alba*), despite a positive self-report	
Positive	293 (72.7)
Negative	110 (27.3)
Probable Bruxism based on pain upon palpation on masseter or temporal muscles, despite a positive self-report	
Positive	84 (20.8)
Negative	319 (79.2)
Probable Bruxism based on frequent headaches, despite a positive self-report	
Positive	156 (38.7)
Negative	247 (61.3)
Probable Bruxism based on tooth wear on enamel or dentin, despite a positive self-report	
Positive	399 (99.0)
Negative	04 (01.0)
Probable Bruxism based on tooth wear on dentin, despite a positive self-report	
Positive	02 (00.5)
Negative	401 (99.5)
Probable Bruxism based on indentation marks on the tongue or *linea alba*, despite a positive self-report	
Positive	192 (47.6)
Negative	211 (52.4)

**Table 6:** Prevalence of Probable Awake Bruxism in adolescents based on different diagnose criteria.

Variables	Frequency (%)
Possible Awake Bruxism	
Positive	208 (51.6)
Negative	195 (48.4)
Probable Awake Bruxism based on positive possible awake bruxism + presence of clinical signs and symptoms (frequent headaches or pain upon palpation on masseter or pain upon palpation on temporal or tooth wear on enamel or tooth wear on dentin or indentation marks on the tongue or *line alba*)	
Positive	207 (51.4)
Negative	196 (48.6)
Probable Awake Bruxism based on positive possible awake bruxism + presence of clinical signs and symptoms of bruxism (frequent headaches or masseter pain upon palpation or temporal pain upon palpation or tooth wear on dentin or indentation marks on the tongue or *line alba*)	
Positive	170 (42.2)
Negative	233 (57.8)
Probable Awake Bruxism based on positive possible awake bruxism + pain upon palpation on masseter or temporal muscles	
Positive	64 (15.9)
Negative	339 (84.1)
Probable Awake Bruxism based on positive possible awake bruxism + frequent headaches	
Positive	108 (26.8)
Negative	295 (73.2)
Probable Awake Bruxism based on positive possible awake bruxism + tooth wear on enamel or dentin	
Positive	206 (51.1)
Negative	197 (48.9)
Probable Awake Bruxism based on positive possible awake bruxism + tooth wear on dentin	
Positive	01 (00.2)
Negative	402 (99.8)
Probable Awake Bruxism based on positive possible awake bruxism + indentation marks on the tongue or *linea alba*	
Positive	104 (25.8)
Negative	299 (74.2)
Probable Bruxism based on clinical signs and symptoms of bruxism (frequent headaches or pain upon palpation on masseter or pain upon palpation on temporal or tooth wear on enamel or tooth wear on dentin or indentation marks on the tongue or *line alba*), despite a positive self-report	
Positive	402 (99.8)
Negative	01 (00.2)
Probable Bruxism based on clinical signs and symptoms of bruxism (frequent headaches or pain upon palpation on masseter or pain upon palpation on temporal or tooth wear on dentin or indentation marks on the tongue or *line alba*), despite a positive self-report	
Positive	293 (72.7)
Negative	110 (27.3)
Probable Bruxism based on pain upon palpation on masseter or temporal muscles, despite a positive self-report	
Positive	84 (20.8)
Negative	319 (79.2)
Probable Bruxism based on frequent headaches, despite a positive self-report	
Positive	156 (38.7)
Negative	247 (61.3)
Probable Bruxism based on tooth wear on enamel or dentin, despite a positive self-report	
Positive	399 (99.0)
Negative	04 (01.0)
Probable Bruxism based on tooth wear on dentin, despite a positive self-report	
Positive	02 (00.5)
Negative	401 (99.5)
Probable Bruxism based on indentation marks on the tongue or *linea alba*, despite a positive self-report	
Positive	192 (47.6)
Negative	211 (52.4)

Most studies in children and adolescents evaluating probable awake and sleep bruxism consider a positive parental- or self-report along with clinical signs/symptoms of bruxism, and in those cases, tooth wear, pain upon palpation on masseter and/or temporal muscle, and jaw-closing muscle pain are the clinical signs/symptoms most evaluated ^5^ ^,^ ^6^ ^,^ ^17^. One population-based study with Brazilian children evaluated probable sleep bruxism based on presence of dental attrition wear in three or more teeth associated or not with parental report of sleep bruxism activity, but authors did not specify the tooth wear severity considered in clinical examination ^17^. In the current study, the prevalence of possible awake bruxism and the prevalence of probable awake bruxism whose diagnosis had been based on self-report along with the presence of any clinical signs/symptoms were the same. The same occurred with possible sleep bruxism and probable sleep bruxism whose diagnosis had been based on self-report along with the presence of any clinical signs/symptoms. Based on those findings, it is important to investigate when clinical signs/symptoms are combined with a positive report, whether the report can become the determinant factor for bruxism diagnosis; therefore, in those cases, clinical examination for bruxism diagnosis would be ‘unnecessary’.

A study found that 17.1% of Dutch adolescents were unaware of sleep bruxism activity and 12.4% were unaware of awake bruxism ^4^. Another Brazilian study found that 80.1% of adolescents who exhibited tooth wear and pain upon palpation on the masseter muscle (clinical signs/symptoms used to diagnose probable sleep bruxism) did not report the behavior ^6^. The diagnosis of probable awake and sleep bruxism based on clinical signs/symptoms is important, especially considering that adolescents might be unaware of the behavior, but the diagnosis criteria must rely on robust and high-quality scientific evidence, in order to be reliable. It is unclear in the literature if awake and sleep bruxism and their different activities (e.g., clenching, grinding, bracing, and thrusting) share the same clinical features. In addition, it is possible that children and adolescents exhibit different clinical signs/symptoms in comparison to adults.

The distinguishment between awake and sleep bruxism was highly encouraged in the first International Consensus and reinforced in its recent publication, where the two distinguished definitions for awake and sleep bruxism were presented ^1^. Prior to those publications, it was very common for researchers to evaluate only ‘bruxism’ in their study ^2^ ^,^ ^3^. Most studies evaluating the diagnosis of possible awake and sleep bruxism in adolescents are based on self-report (4,18-20). One recent study with 8- to 11-year-olds from Brazil also considered the frequency of occurrence of awake bruxism, and found a similar result, with 38.6% reporting ‘sometimes’ grinding/clenching their teeth and 7.1% reporting ‘often’ ^18^. Other studies found a lower prevalence of possible awake bruxism, ranging from 4.1% to 19.2% in adolescents from different countries with different ages ^19^ ^,^ ^20^. One study from Canada evaluated possible awake bruxism with a diagnosis based on parental report and found a prevalence of 12.4% in 7- to 17-year-olds ^7^.

The prevalence of possible sleep bruxism found in our study (considering grinding, bracing, and thrusting activities) was higher in comparison to previous studies. Other studies basing the diagnosis on adolescents’ self-report found a prevalence ranging from 7.6% to 18% ^4^ ^,^ ^19^ ^,^ ^20^. The diagnosis of possible sleep bruxism can also be done based on parental report, as presented by a Canadian study involving 7- to 17-year-old adolescents, with a 15% prevalence ^7^, and 22.2% prevalence in a Brazilian study with 11- to 14-year-olds ^21^. The questions used for diagnosis in those studies referred specifically to ‘grinding the teeth and/or clenching the jaws’ during sleep ^4^ ^,^ ^7^ ^,^ ^19^ ^,^ ^21^, not considering activities, such as bracing and thrusting. The non-evaluation of other bruxism activities could explain the differences in prevalence, once the prevalence of self-reported sleep bruxism grinding activity in the current study is similar to the prevalence reported by previous studies. Another Brazilian study found a prevalence of possible sleep bruxism of 33.4% in 13 to 15-year-old adolescents ^22^, a result similar to the one found in the current study. Authors based their assessment on adolescents’ self-report or parental report of ‘audible sounds of teeth grinding’ while asleep ^22^; other activities of bruxism were not considered. The diagnosis based on both self-report and parental report might explain the higher prevalence of possible sleep bruxism grinding activity, since adolescents might be unaware of sleep bruxism occurrence ^4^. 

Self-report of frequent headaches and pain upon palpation on masseter and temporal muscles were the clinical signs/symptoms associated with possible sleep and awake bruxism. Previous studies found similar results. A Study with Dutch individuals aged 10 to 22 years found that self-report of pain or tense feeling in the jaws upon wakening in the morning was associated with self-reported sleep bruxism and self-report of orofacial pain was associated with awake bruxism ^4^. A recent systematic review found that headache was one of the clinical signs/symptoms most prevalent in children with sleep bruxism, along with primary canine wear and dental wear ^5^. However, the studies included in the systematic review had a focus on children in primary dentition and had a low to very low certainty of evidence, highlighting the need of new studies with a more robust method ^5^. In a polysomnographic study with 16 adolescents with definite sleep bruxism, all participants reported frequent headaches at some period of the day ^23^, reinforcing the possible link between bruxism activity, headache, and muscle pain. 

The current study has some limitations that must be pointed out. First, the cross-sectional design of the current study does not allow one to conclude that the clinical signs/symptoms evaluated were caused by bruxism occurrence. Longitudinal studies evaluating bruxism occurrence and clinical features through dentition development are essential to determine causality. Due to the lack of a validated questionnaire or instruments to evaluate bruxism self-report among adolescents, authors developed the questions used in the current study. This might make it difficult to compare the results of the present study with the findings of other studies, in which other questions and methods to assess bruxism activity had been used. To minimize this limitation, we included the questions used to evaluate bruxism activity in the methods, so that researchers assessing different populations can use the same questions or compare their results when using similar questionnaires.

The polysomnography and electromyography are considered the gold standard to diagnose sleep bruxism and awake bruxism, respectively ^1^; however, those diagnosis tools have a high cost and limited availability, making the applicability in epidemiological studies with big samples and daily clinical practice unfeasible. Unfortunately, no study evaluating the agreement between self-report, clinical features, and instrumental assessment of bruxism in adolescents has been identified thus far. The agreement between bruxism self-reported measures and electromyography devices in adults are low to modest, and authors encourage researchers and clinicians to consider other clinical methods along with patient’s report ^24^; however, further investigation is necessary to determine which clinical features should be considered, especially in youngsters.

This exploratory study aimed to evaluate the prevalence of probable awake and sleep bruxism considering different diagnostic criteria. A high prevalence variability for both probable awake and sleep bruxism in the same sample was found. This means that the definition of diagnostic criteria for bruxism will impact directly on its prevalence, and consequently on results of associated factors. Epidemiological studies can be helpful in identifying distribution and factors underlying the source of a condition, being a useful tool for the understanding of diseases and health conditions ^25^. The results found in this exploratory analysis can serve as a basis for future studies, in which sensitivity analysis can be performed to compare results with different diagnostic criteria. This high variability of probable awake and sleep bruxism according to different diagnosis in the same sample makes these criteria questionable in adolescents and reveals the importance of future studies evaluating the clinical signs/symptoms of bruxism and its prevalence in adolescents in permanent dentition. The results reported in the current study also highlight the importance of a detailed anamnesis and clinical evaluation of bruxism behavior in young patients and for research purpose.

As a conclusion, a high inconsistency was found for the prevalence of probable awake and probable sleep bruxism in adolescents based on different diagnosis criteria according to the International Consensus. The wide range in probable awake bruxism and probable sleep bruxism prevalence were influenced by the different clinical sings/symptoms used as diagnosis criteria. Frequent headaches (three or more times a week) and pain upon palpation on masseter and temporal muscle were associated to possible awake bruxism and possible sleep bruxism among adolescents.
